# Quantitative Analysis of Staphylococcus Enterotoxin A by Differential Expression of IFN-γ in Splenocyte and CD4^+^ T-Cells

**DOI:** 10.3390/s140508869

**Published:** 2014-05-20

**Authors:** Reuven Rasooly, Bradley J. Hernlem

**Affiliations:** Western Regional Research Center, Agricultural Research Service, U.S. Department of Agriculture, Albany, CA 94710, USA; E-Mail: bradley.hernlem@ars.usda.gov

**Keywords:** staphylococcal enterotoxins, interferon-gamma, active toxin detection, CD4^+^ T-cell, splenocytes

## Abstract

*Staphylococcus aureus* is an important bacterial pathogen that produces a range of Staphylococcal Enterotoxins (SEs) which cause gastroenteritis and superantigen activation of T cells, the mechanism of which is not well understood. The ability to rapidly detect and quantify SEs is very important in order to learn the causes of staphylococcal outbreaks and to stop similar outbreaks in the future. Enzyme-linked immunosorbent assays (ELISAs) have been developed for detection of several SEs. However, these immunological methods cannot distinguish between active and inactive toxin. It is known that interferon-gamma (IFN-γ) expressed in response to stimulation by SEs contributes significantly to the pathogenesis of *S*. *aureus* infection. Nonetheless, the cellular source of IFN-γ is still unclear and the contributions of the specific splenocyte types. In our effort to understand the immunologic response to Staphylococcal Enterotoxin A (SEA) exposure, we studied IFN-γ production in mouse splenocytes. We demonstrated that short term *ex vivo* exposure of splenocytes or primary naïve CD4^+^ T-cells to biologically active SEA induces differential expression of IFN-γ mRNA in a time and dose dependent manner and the expression levels reflect the levels of IFN-γ secreted protein. Positive isolated CD4^+^ T-cells accounted for only 10% of IFN-γ production. We also demonstrate that expression of IFN-γ can be used for rapid quantitative analysis of active SEA with a detection limit of 1 ng/mL.

## 1. Introduction

*Staphylococcus aureus* is a leading bacterial pathogen of concern and a major cause of clinical infections and foodborne illnesses [1]. The pathogenicity of this bacterium is manifested through the production of enterotoxins (SEs), twenty-one of which have thus far been identified. The SEs target the gastrointestinal tract and also behave as superantigens in immune system response. These responses have been shown to be linked [2,3]. Mutation studies demonstrate that the loss of superantigenic activity is correlated with a similar loss in enterotoxicity [4]. The ability to rapidly detect and quantify active SEs is very important in order to learn the causes of staphylococcal outbreaks and to stop similar outbreaks in the future. Thus it is essential to develop assays that distinguish the biologically active form of the toxin, which poses a threat, from the inactive form which poses no threat. SEA has been associated with three quarters of staphylococcal outbreaks and one such incident sickened 13,420 individuals in Japan when powdered skim milk was determined to be contaminated with SEA [5,6]. In response to the need for improved methods to detect active SEs, we developed and reported a non-radioactive splenocyte cell-based assay which detects active SEA in food after 48 h [7] with greater sensitivity then alternate methods which rely upon *in vivo* response in monkey or kitten.

It has been shown that interferon-gamma (IFN-γ), a proinflammatory cytokine that keeps viruses from replicating and up-regulates the expressions of other disease-fighting agents, contributes significantly to the pathogenesis of *S*. *aureus* infection which causes toxic shock syndrome. However, the relative contribution of CD4^+^ T-cells subsets to IFN-γ response during SEA induced proliferation has remained largely undetermined. In addition, it in unknown whether CD4^+^ T-cells alone produce differential quantities of IFN-γ after SEA exposure. In this study we identify that only 10% of IFN-γ is produced by naïve CD4^+^ T-cells and IFN-γ is differentially expressed in splenocyte and CD4^+^ T-cells after SEA exposure. The result shows that the levels of IFN-γ mRNA and its protein expression and secretion after SEA exposure can be used for detection and quantification of biologically active SEA.

## 2. Experimental Section

### 2.1. Chemicals and Reagents

SEA was obtained from Toxin Technology (Sarasota, FL, USA). CD4^+^ T-cell positive isolation kits were obtained from Invitrogen (Carlsbad, CA, USA). Mouse Cytometric Bead Array (BD Catalog No. 560485) for capture and detection of IFN-γ was obtained from BD Biosciences (San Diego, CA, USA).

### 2.2. Splenocyte Isolation

Spleens from C57BL/6 female mice were aseptically removed and disrupted using a syringe and needle in Russ-10 cell culture medium (made by combining 450 mL of RPMI 1640 medium without glutamine (Gibco/Life Technologies, Carlsbad, CA, USA), 50 mL fetal bovine serum (Hyclone, Logan, UT, USA), 5 mL 200 mM glutamine (Gibco), 5 mL antibiotic-antimycotic (Gibco; containing penicillin, streptomycin, and fungizone), 5 mL nonessential amino acid mix (Gibco), 5 mL sodium pyruvate (Gibco), and 0.25 mL of 100 mM beta mercaptoethanol (Sigma, St. Louis, MO, USA). Cells were centrifuged at 200× *g* at 4 °C for 10 min. Red blood cells were then lysed by adding red cell lysis buffer (0.15 M NH_4_Cl, 10 mM KHCO_3_, 0.1 mM Na_2_EDTA). Cells were again centrifuged and resuspended in Russ-10 medium, and viable cells were counted using trypan blue and a hemocytometer.

### 2.3. Positive Isolation of Murine CD4^+^ T-Cells

Using fresh splenocytes, murine CD4^+^ T-cells were isolated using a positive isolation method (Dynabeads Mouse CD4 L3T4 Isolation Kit, Invitrogen/Life Technologies, Carlsbad, CA, USA), according to the manufacturer's instructions. Briefly, splenocytes were resuspended in isolation buffer (PBS supplemented with 0.1% BSA and 2 mM EDTA) at a concentration of 1 × 10^7^/mL, and incubated with washed Dynabeads (25 μL of Dynabeads per 10^7^ cells) for 20 min on ice with gentle rotation. After incubation, the cells and Dynabeads were placed on a magnet for 2 min. The supernatant was removed and the bead-bound cells were washed with isolation buffer three times. The bead-bound cells were resuspended in Russ-10 media (10^7^ cells per 100 μL of media) and DETACHaBEAD mouse CD4 was added (10 μL per 10^7^ cells) and incubated for 45 min with gentle rotation at room temperature. The detached cells were washed three times and resuspended in media.

### 2.4. Quantitative RT-PCR Analysis

Quantitative RT-PCR (qRT-PCR) analysis for the early detection of SEA that induce IFN-γ was done as described previously [8]. The forward primer used for IFN-γ, was 5′-CATTGAAAGCCTAGAAAGTCTG-3′, and the reverse primer used was 5′-CTCATGAATGCATCCTTTTTCG-3′. The housekeeping gene hypoxanthine-guanine phosphoribosyl transferase (Hprt) forward primer 5′-GTTGGATACAGGCCAGACTTTGTTG-3′ and the reverse primer used was 5′-GAGGGTAGGCTGGCCTATAGGCT-3′.

### 2.5. Cytokine IFN-γ ELISA

IFN-γ was measured in cell culture supernatants by capture ELISA Abs, purified standards, and protocols suggested by the manufacturer BD Biosciences Catalog No. 555138.

### 2.6. Statistical Analysis

Statistical analysis of ELISA data was performed using SigmaStat 3.5 for Windows (Systat Software, San Jose, CA, USA). Multiple comparisons of the spiked culture media were made. One-way analysis of variance (ANOVA) was used to compare control unspiked media with media that contained increasing concentrations of SEA. The experiments were repeated at least three times and results with p < 0.05 were considered statistically significant.

### 2.7. Flow Cytometry

IFN-γ in splenocyte and CD4^+^ T-cells was quantified using a Mouse Cytometric Bead Array (BD Catalog No. 560485) for capture and detection of IFN-γ, using the method specified by the manufacturer (BD Biosciences). Data were collected using a modified FACS Vantage SE (BD Biosciences) having a Cobolt Calypso^TM^ 491 nm (blue), 100 mW primary laser (Cobolt AB, Stockholm, Sweden) and a Cube^TM^ 640 nm (red), 40 mW secondary laser (Coherent Inc., Santa Clara, CA, USA). Bead populations targeting IFN-γ, were discerned by fluorescence intensity in a channel excited by the red laser and selected using a 676/29 nm bandpass filter (Semrock, Rochester, NY, USA). Blue laser excited R-phycoerythrin (PE) fluorescence reported cytokine intensity and was quantified using a 585/42 nm bandpass filter. Data were initially analyzed using FlowJo software (TreeStar Inc., Ashland, OR, USA) and subsequently with SigmaPlot 11.0 software (Systat Software, San Jose, CA, USA) for statistical analysis and fit to standard data.

## 3. Results and Discussion

### 3.1. IFN-γ is Differentially Transcribed after SEA Exposure

**Figure 1.**
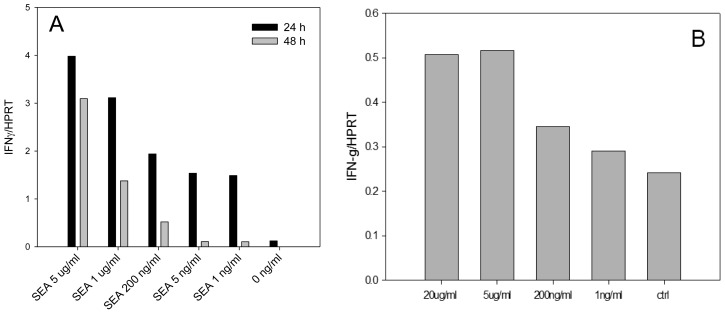
Biologically active SEA induces IFN-γ mRNA levels in dose dependent response. IFN-γ/HPRT mRNA ratio was measured after incubation of splenocytes for 24 and 48 h (**A**) or positively selected mouse naïve CD4^+^ T-cells for 24 h; (**B**) with decreasing concentrations of SEA.

Enterotoxins were shown to induce high levels of IFN-γ secretion and significantly contribute to the pathogenesis of *S*. *aureus* infection. In order to develop an assay for early detection of active SEA we examined if SEA is able to induce IFN-γ mRNA in dose dependent manner in splenocytes. We also investigated if SEA is able to induce IFN-γ mRNA in positively selected naïve CD4^+^ T cells in the absence of other splenocyte cells subtypes. Our quantitative real-time PCR (qRT-PCR) (Figure 1) reported as relative to the commonly used housekeeping gene HPRT, shows that IFN-γ mRNA is differentially expressed and that biologically active SEA can be detected at a concentration of 1 ng/mL after 24 h.

The results shown in Figure 1 illustrate that SEA was able to induce IFN-γ mRNA in dose dependent manner on both splenocyte and positively selected mouse naïve CD4^+^ T-cells. Using positive isolation, the concentration of CD4^+^ T-cells was enriched from an average 22% of splenocytes to 95%. However, the results show that this enriched subpopulation of cells, in the absence of other splenocyte subtypes, exhibited only 10% SEA induced IFN-γ transcription compared with non-selected splenocytes.

### 3.2. IFN-γ Expression after Stimulation of Splenocytes or CD4^+^ T-Cells with SEA

**Figure 2.**
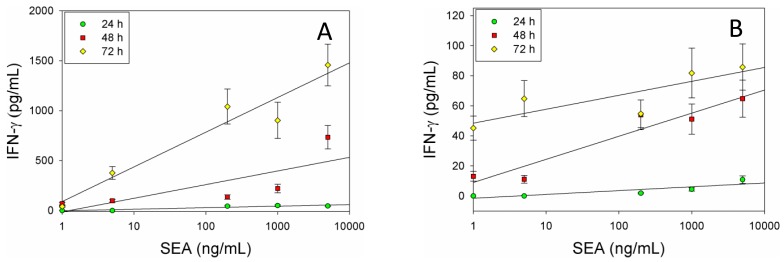
Flow cytometric analysis of IFN-γ protein secretion by splenocytes (**A**) or CD4^+^ T-cells (**B**) after exposure to biologically active SEA. Splenocytes were spiked with increasing concentrations of SEA. After 24, 48 or 72 h of stimulation with SEA, IFN-γ protein secretion was measured by flow cytometry. Linear regression fits to the data are shown and error bars represent standard errors for each set of data points.

The molecular data shows that IFN-γ expression is upregulated in dose dependent manner at the transcriptional level in CD4^+^ T-cells and splenocytes treated with SEA. To verify the qRT-PCR results and to demonstrate that this upregulation enhances the kinetics of IFN-γ protein expression, after stimulation with SEA we incubated splenocytes or positive isolated CD4^+^ T-cells with SEA for 24, 48, and 72 h. After intracellular staining, kinetic IFN-γ production was assessed by flow cytometry. Flow cytometric analysis shows that *ex vivo* stimulation of splenocytes (Figure 2A) or primary naïve CD4^+^ T-cells (Figure 2B) with SEA for 48 and 72 h induces the expression of IFN-γ in dose dependent response on both splenocyte and positively selected mouse naïve CD4^+^ T-cells. The data suggest that there is a correlation between the levels of IFN-γ mRNA and the secretion of IFN-γ protein. As shown in Figure 2B SEA induced IFN-γ protein levels were similarly reduced of IFN-γ in the CD4^+^ T-cell enriched subpopulation. The data also show that the production of IFN-γ increased over time and SEA can be detected at a concentration of 1 ng/ml.

### 3.3. Detection of Biologically Active SEA by ELISA

**Figure 3.**
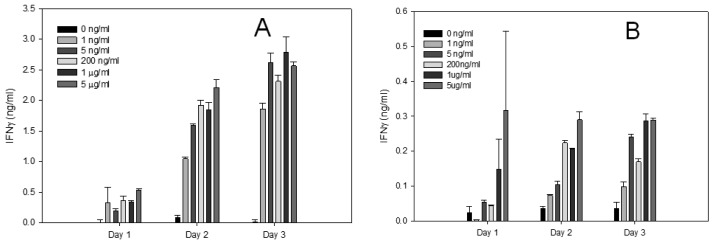
Quantification of biologically active SEA using ELISA assay. Increasing concentrations of SEA was incubated with splenocytes (**A**) or CD4^+^ T-cells (**B**). After 24, 48 or 72 h of stimulation with SEA, IFN-γ protein secretion was measured by ELISA assay. Error bars represent standard error.

Although our flow cytometry results were rapid and sensitive, using this analytical method requires expensive equipment and skilled personnel. To overcome this aspect, we used an ELISA test that measured the secreted IFN-γ protein produced from splenocytes and positive isolated CD4^+^ T-cells (Figure 3). The ELISA assay is relatively simple, inexpensive and practical for simultaneous testing of large number of samples. The ELISA results confirmed that the levels of IFN-γ produced by CD4^+^ T-cells were lower than IFN-γ protein produced by splenocytes. The results demonstrated that active SEA induces expression of IFN-γ in a dose dependent response and SEA can be detected at a concentration of 1 ng/mL after 48 h.

## 4. Conclusions/Outlook

The current methods to detect active SEA, associated with 75% of food borne staphylococcal outbreaks [9], are an *in vivo* monkey or kitten bioassay [10,11]. However, these procedures have low sensitivity and poor reproducibility. Enzyme-linked immunosorbent assays (ELISA) and mass spectrophotometry (MS) have been developed for detection of several SEs [11,12], but these immunological methods cannot distinguish between active and inactive forms of the toxin. Previously, we developed a sensitive assay that can detect and quantify active SEA. While this assay, which measures BrdU incorporation into cellular DNA, is very sensitive with good reproducibility, it requires 48 h incubation [7]. It was found that a subtype of mouse naïve CD4^+^ T cells expresses MHC (major histocompatibility complex) class II molecules on their cell surface and these CD4^+^ T cells can perform the role of both antigen presenting cell and T cells. Thus, they are able to present SEA to themselves or neighboring CD4^+^ T cells via MHC class II. We showed that in the absence of nearly all other splenocyte cell types there was no decreased SEA induced proliferation of naïve CD4^+^ T cells compared to proliferation levels of all splenocytes [13]. It has been shown that IFN-γ, which is expressed in response to stimulation by SEs, contributes to the pathogenesis of *S*. *aureus* infection. However, it is still unclear the cellular source of IFN-γ. CD4^+^ T cells are believed to be the primary cellular source of IFN-γ during SEA stimulation. Our results show that there was less induction of IFN-γ by SEA in naïve CD4^+^ T cells compared to splenocytes. After stimulation with SEA, CD4^+^ T-cells accounted for only 10% of total IFN-γ production compared to splenocytes. This result suggests that CD4^+^ T-cells were not the only source of IFN-γ production in splenocytes. It has previously been shown by [14] that in CD4^+^ T cell-deficient mice, IFN-gamma production was due to CD8^+^ T cells. It has been shown that neutrophils are a source of IFN-γ during acute *Salmonella* colitis [15]. Our results show that IFN-γ mRNA and secretion of IFN-γ protein are differentially expressed after exposure to SEA. The result suggests that induction of IFN-γ mRNA expression and protein production can be used for quantitative analysis of SEA. The result also suggests that the assay can distinguish between biologically active form of SEA, which poses a threat, and the inactive form which poses no threat.

Employed alone, the assay cannot distinguish SEA from other unknown toxins or elicitors of IFN-γ. Furthermore, as when analyzing complex matrices such as food, the induction of IFN-γ by SEA in food may require prior selective isolation of SEA from the food matrices and subsequent analysis to address the concern about the effect of the matrix and possible confounding elicitors. We have previously demonstrated that by concentrating the toxin from a large volume and disassociation of SEA using immunomagnetic beads, we were able to improve the specificity and sensitivity to detect 20 pg/mL of active SEA [7].

Finally, while it is expected that a similar assay may be performed using human PBMCs, the use of splenocytes from mice is a more widely and practical method. The use of PBMCs from healthy human donors requires approval by an institutional review board and the need of a phlebotomist, both of which add complexity. Furthermore using PBMCs from human is not practical for a clinical method because unlike mice, human healthy donors are not always available.

## References

[1] Dinges M.M., Orwin P.M., Schlievert P.M. (2000). Exotoxins of Staphylococcus aureus. Clin. Microbiol. Rev..

[2] Hui J., Cao Y., Xiao F., Zhang J., Li H., Hu F. (2008). Staphylococcus aureus enterotoxin C2 mutants: Biological activity assay in vitro. J. Ind. Microbiol. Biotechnol..

[3] Hu D.L., Omoe K., Sashinami H., Shinagawa K., Nakane A. (2009). Immunization with a nontoxic mutant of staphylococcal enterotoxin A, SEAD227A, protects against enterotoxin-induced emesis in house musk shrews. J. Infect. Dis..

[4] Harris T.O., Grossman D., Kappler J.W., Marrack P., Rich R.R., Betley M.J. (1993). Lack of complete correlation between emetic and T-cell-stimulatory activities of staphylococcal enterotoxins. Infect. Immun..

[5] Asao T., Kumeda Y., Kawai T., Shibata T., Oda H., Haruki K., Nakazawa H., Kozaki S. (2003). An extensive outbreak of staphylococcal food poisoning due to low-fat milk in Japan: Estimation of enterotoxin A in the incriminated milk and powdered skim milk. Epidemiol. Infect..

[6] Ikeda T., Tamate N., Yamaguchi K., Makino S. (2005). Mass outbreak of food poisoning disease caused by small amounts of staphylococcal enterotoxins A and H. Appl. Environ. Microbiol..

[7] Rasooly R., Do P.M. (2009). *In vitro* cell-based assay for activity analysis of staphylococcal enterotoxin A in food. FEMS Immunol. Med. Microbiol..

[8] Rasooly R., Kelley D.S., Greg J., Mackey B.E. (2007). Dietary trans 10, cis 12-conjugated linoleic acid reduces the expression of fatty acid oxidation and drug detoxification enzymes in mouse liver. Br. J. Nutr..

[9] Vernozy-Rozand C., Mazuy-Cruchaudet C., Bavai C., Richard Y. (2004). Comparison of three immunological methods for detecting staphylococcal enterotoxins from food. Lett. Appl. Microbiol..

[10] Bergdoll M.S. (1988). Monkey feeding test for staphylococcal enterotoxin. Methods Enzymol..

[11] Bennett R.W. (2005). Staphylococcal enterotoxin and its rapid identification in foods by enzyme-linked immunosorbent assay-based methodology. J. Food Prot..

[12] Dupuis A., Hennekinne J.A., Garin J., Brun V. (2008). Protein Standard Absolute Quantification (PSAQ) for improved investigation of staphylococcal food poisoning outbreaks. Proteomics.

[13] Rasooly R., Do P.M., Hernlem B.J. (2011). Auto-presentation of Staphylococcal enterotoxin A by mouse CD4^+^ T cells. Open J. Immunol..

[14] Caruso A.M., Serbina N., Klein E., Triebold K., Bloom B.R., Flynn J.L. (1999). Mice deficient in CD4 T cells have only transiently diminished levels of IFN-gamma, yet succumb to tuberculosis. J. Immunol..

[15] Spees A.M., Kingsbury D.D., Wangdi T., Xavier M.N., Tsolis R.M., Bäumler A.J. (2014). Neutrophils are a source of gamma Interferon during acute Salmonella enterica serovar typhimurim colitis. Infect. Immun..

